# Dogmas and Fashions in Practice

**Published:** 1905-10

**Authors:** E. C. Cartledge

**Affiliations:** Atlanta, Ga.


### DOGMAS AND FASHIONS IN PRACTICE.

E. C. CARTLEDGE, M. D., ATLANTA.

The other day I read in Johnston's Manual of Botany, that in the green leaves of plants, not only was starch built up from the water ( $H_2O$ ) of the plant and the $CO_2$ of the air, but that proteids ( containing N ) were also manufactured there in some way not yet comprehended. This puts one to thinking, and suggests many problems, some of which we think we know.

It would appear no great question as to how the leaf built up proteids into itself and its plant, since the air is 3-4 N by volume. But the companion thought is, what attracts us; *i. e.*, why can not and does not the humany economy get N in that way? And how much the N may have to do more than to simply dilute the $O_2$ we breath we may not know—whether it may not find some entrance into our circulation and tissues. We are informed that muscle (the N tissues) can be built up from C H O foods. Whence comes the N? Somebody may discover for us some way that we swallow, while eating or not, an amount of air and N that is appropriated. I know a man who says he relieves a stomach discomfort, by swallowing a good bulk of air into it. The most brawny, red cheeked and headed little lad of about five years of age I ever saw ate no meats or eggs and only bread stuffs, mostly rice and hominy.

These two paradoxical facts were so very pronounced, that they were frequently remarked upon by family and friends.

We all are sure that iron is good to enrich the blood. I doubt it. More surely for many years past have chemists and the scientific world used the atomic theory, as the basis upon which rested the science of chemistry; and science deals with things known.

We have recently been taught to unlearn this teaching and many of its applications which our brows did ache and toil to learn. The ion, or electron, now takes the place of the atom.

This same ebb and flow, fashion and fad, in our practice of medicine, even more than in some of the more exact sciences, makes us feel in doubt and like proving all things, which is a very hard thing to do. Many medicine manufacturers are cunning enough to deceive us if we would accept their suggestion of how valuable is their remedy in the convalescing stage of various ailments, the medicine receiving credit for doing what nature does in strides and bounds, when the human physiology first escapes the trammels of disease. We smile at how good certain new remedies are in gastric and intestinal troubles combined with Bism, Pepsin and Aro. Chalk Po.

How hard was the lesson with my first patient when I learned that soda salicylate nor any other drug would cure rheumatism. With something of less chagrin, I cured (?) my next similar case with three doses from a pint bottle, which my good grateful patient left out where I could be reminded of giving her a large $1.50 bottle, when I knew three doses would cure her. There comes to my mind four cases of rheumatism : One took very best medicine scientifically, but ate a large piece of sweet cake three days before she died. Another was forced fed at regular intervals, scientifically under professional hospital nursing, till her tongue was brown coated, skin yellow and she died. Another reported here one evening, was operated repeatedly and treated medically for about a year when it was learned that the trouble and wisdom of treatment related to the intestinal tract. The fourth case, with a prospect as bad as the worst, was cured by the absolute detail of bowel, skin and kidney elimination and food positively limited in kind and quantity to the small point of sure assimilation, etc.

A few years ago scarlet fever cases and other fevers died, being allowed only a teaspoonful of water at infrequent intervals. We all hear our elders speak of that former fashion of treatment and how the famished sick recklessly stole to the bucket and drank their fill and recovered from that good hour. Now we receive a three or four days' lecture in our college class just on water and know not how to cope with disease without $H_2O$ as a universal eliminant, etc. And yet we may not, by every precedent, have passed and surpassed our equally honest fathers so far, but that this decade and age, golden to us, may become the dark age to those after us. There still remains in the minds of old people and the uninformed, that scarlet fever is a very fatal disease, due to the way our profession used to kill them till those who stole to the bucket of water revealed the truth. These were in the generally intellectual days of Henry Clay, Daniel Webster and John C. Calhoun.

I will pass blood-letting by, once as common as is giving calomel now. And that reminds us that several years ago, some one high in authority (perhaps because he wrote a book like Hobart A. Hare in which he devotes great length to the value of milk in typhoid), anyway, some one high in authority tested and proved that calomel did not act on the liver. The profession guessed it must be true and calomel became very unfashionable for a long season. The aunt Jemimies and some Eclectic brothers still think calomel softens the bones, causes rheumatism and is inferior to Leptandrin and Euonymin.

Opium has been found in ancient Egyptian tombs. Hippocrates recommended arsenic for cancer. Hernias were operated on 350 B. C. The ancients used stramonium and belladonna and from wild lettuce obtained an active principle having much the effect of opium. After centuries, history repeats itself and just now, once again is coming in vogue, what the Greeks and Romans practiced in hydrotherapy, gymnastics and open-air treatment.

Organo-therapy is passing in review the second time at least, and I'm uninformed if the orchitic lymph from bulls and goats has done more than that the mind of the patient, and doctor too, has been stimulated and invigorated and rejuvenated.

The country swain used to come into my drugstore in the mountains and buy love powders to give his unresponsive loved one. He received powdered pepsin—Fairchild's best scales—and it usually worked well and won her heart and hand—occasionally needing a "refill." You may have heard of the absent-minded doctor who left his thermometer under his patient's arm, remaining there till doctor came back the following day to see him. The patient reported that he didn't know what that little medicine under his arm was, but it had done him a power of good.

Dr. Harris, discussing alcohol one evening here at our meeting, quoted from some old book, pretentious in size but obsolete, where jalap and sulphur, I believe, were heralded as specific for malarial fever.

A message came for me to visit a patient today or tomorrow, I came on the morrow. She was up and well, but was nearly dead she said on the day before with aching severely and high fever—lagrippe. The result was nature's but my credit if I had been there. Only my lagrippe records are two or three days. The only doctor to whom I confided how I hoped to cure uterine or other cancer, by local medical application, taunted me with being simple-minded. Within the month a brother physician told me of his greater encouragement in that hope than my experience had offered to me.

Some time ago the assistant professor of Electro-therapeutics in the New York Post-Graduate Medical School located in our community and was tabooed by us as a quack. Since we ran him back home there are more than a dozen of us who have outfits like his and are trying to do the same kind of work, using his book and periodical publications, and have discovered how very poorly we could do without the help of our electric and X-ray machines. In like fashion was Harvey tabooed for his heresy that arteries contained not air, but blood.

And fifty years ago when Drs. Henry MacCormac and George Bodington proclaimed cold fresh air and a liberal diet for consumptives they were ostracised by their stereotyped brethren.

And till now our human nature remains rather much the same sometimes, as when in Tristate medical meeting here not long ago one brother proclaimed most 100 cases of appendicitis cured without operation and another budding young surgeon declared with great emphasis that every case ought to be operated on on the spot and the latter proposition received hearty encore from the junior constituency.

I can imagine that the irony of their dogmatism decreed that Bro. Conservative had to operate on his next three cases and Bro. Extremist had about six cases to recover before he could prepare for the operation. Very little of truth is developed in such discussion for the honest hungry-minded doctor who has at heart only to serve his patient best and not self-exploitation. May we all realize that weight of awful responsibility that is ours with sickness, life and death.

I may say that I hate whisky for the great blight and blast, wreck and ruin it has wrought against body and soul of man.

Ever suspicious toward it, I'm trying to watch and be fair if ever I see it do any real good in sickness.

It must say it makes me glad to believe its use as a medicine is a fad that is about past. I have about as many reasons for believing it universally bad as I have that air and sunshine are universally good. Some people do very well without much outdoor life of air and sun apparently and just as apparently without harm do some people, sick cr well, take more or less whisky.

I question if giving any preparations of iron have ever done any good. Don't side against me, but listen willingly enough to hereafter try for yourself—always for the sake of truth.

From an old note-book I find :

1903. June 23. — Elix Lactopeptine—Iron & Gent. at meal time to Mrs. L. Hall. Constipated her, but regular now, since stopped it.

Few days ago. — Mrs. L. M. says, "Oh, why does Dr. E. always insist on giving me Iron? He knows I can't take any kind of Iron."

" — G. V., child 4 yrs. old, Diphtheria, took ℞ Iron, Glyc. & Chlorate Potash, which put her bowels full of foul gas and curded milk with aggravated symts.

June 29. — Miss F. G. couldn't take Gude Peptomang or Tr.Ferri from Dr. J. L. C. or myself.

" — Mrs. E. B., a convalescing eclampsia, Tr. Ferri gtt xv tid. Temperature relapsed to 103, tympanites & autoinfection.

1905. May. — Mrs. P. at Hosp., was severely constipated by taking Syr. Iodide Iron.

My personal experience was rather tragic to me. I was sick and weak with several months in bed from Bright's disease and could only talk in monosyllables. All who knew my case called it Bright's disease, and I'm sure I had every classical symptom except the test for urea may never have been made, and that is by far the most important, according to my every experience. As for albumen, and especially casts, they are found, granular and epithelial, in most any febrile condition of typhoid, pneumonia, la grippe, or else, where temperature reaches above 102°.

In the routine of a hospital service they are usually quite insignificant. One night after giving me my nightly enema my mother said to me that my stools looked very black.

Happy thought that came to me that that iron in Basham's mixture was what kept my bowels rolling and rumbling with foul gases and no food being digested. The doctor agreed that I might leave it off, and in three days I was so improved I hoped I might live. The next happy event for me was when Miss M. K. begged me to take the recipe that cured her father, himself a physician, and others, she said, of Bright's disease.

She would not be dissuaded, and when I saw the recipe was nux vomica, phosphoric and aro. sulph. acids I took it thinking that the acids might do my liver good, and feeling that my liver was at least *particeps criminis* in my condition.

There was the source of my kidney trouble, and recipe gave me the next great step up.

Then the sun and good air of outdoor life, which are good not alone for consumption, but quite a universal panacea. Tincture of iron is least objectionable, I think, because of the valuable H Cl in it. But I can not take it, Blaud's pill, nor any of the so-called peptonates of iron.

Anyway, the apparent anemia of Bright's disease is partially, I think, a deranged vasomotor nerve supply affecting the cutaneous circulation; for they may change from pale to pink within a few minutes, a frequently varying complexion.

Bright's disease must be chiefly a symptom or secondary disease because it must be caused by irritating impurities coming to the kidneys through the circulating blood, and the thing to treat must be the source of this bad blood supply, usually some mal-assimilation of food or drink. I've seen endometritis do it. Alcohol might and does circulate as alcohol in the blood and do it. I quote Dr. Todd: "Find out the cause of this effect, or rather say, the cause of this defect, for this effect, defective, comes of cause."

There is no more reason for treating the kidney in diabetes or acute Bright's disease than in treating dropsy merely as dropsy. "Bright's disease is as curable as consumption is. It used to be considered as incurable as consumption."

But we were speaking of iron. There are only thirty or forty grains of iron in the human body, and a very few lunches of green salads, fruits and cereals will furnish that supply. Quite all of our foods contain more or less iron. Our only reason for thinking we need iron is that by means of it the red blood conveys oxygen. I suspect 'twould be a much more effectual fad to feed our anemic patients on butter, cream, glycerine, cod liver oil and such to supply fat to bone marrow where red blood corpuscles are built up. A very scant amount of bone marrow is present in anemias I would imagine.

A fashion taught, how much practiced, I do not know, in childbirth, is to hold the head back during the last few labor pains to allow the perineum time to stretch. My experience is that I have not muscular power to hold it back in exactly those cases that need it. There is no place to press nor method of pressure that, in some degree, does not hinder the blood supply there. Also, pressing upon the advancing head tends to destroy the caput succedaneum and elongated lapped condition of head bones present and to make head rounder and of increased diameter to the more easily tear the perineum.

So I use chloroform very happily to retard and forceps to hasten labor. I have used ergot, strychnine and quinine, but they didn't seem to properly discriminate in their effect upon the muscular tissues thereabouts and the tears were larger than I thought they should have been.

Next, with pneumonia in my mind, I remember how Dr. Todd, (whom we shall always love for his own attractive personality and as one of our fathers and philosophers in medicine), was wont to tell the class to never overload or urge a tired, sick horse up a steep hill with a big load on; that extremes meet, exhaustion following over-stimulation.

Therefore, I can not understand to use strychnine and digitalis in struggling pneumonia hearts, but rather veratrum to quiet and aconite to soothe the tempestuous wild beating. I've seen the difference and 'twas perhaps a hundred per cent. in results. Poultices, very hot, and blisters, large and free, added their noble effect at times, though these are now used only by the old-fashioned doctors, I hear.

I've seen no amount of drugs stimulate a failing organic heart as much as supine, perfect rest could have saved it, and he died.

It's a pleasure to hear Dr. Howard Kelly and such experienced men, whom we trust and delight in, cry out to the ears of thousands who listen to them against the needless sacrifice of healthy ovaries.

Pelvic massage was exploited ten years ago, but passed quickly.

It is hard for us all not to be faddist, each in his own peculiar development and specialized views, as for instance, as before remarked on this floor: Headaches are prone to be nervous to the nerve specialists, eye fault and glasses to the oculist, neuralgic from teeth or antrum to the dentist, to the digestive tract says the stomach specialist. The old family doctor, general practician, discovers some combination of these influences the cause. I've recently seen two gonorrheal pus tube cases get well where I had advised strongly an operation.

With what great and rare delight have we seen Dr. Nicolson save feet, hands and limbs that were crushed and mutilated, bone and soft parts into a pulpy mass sometimes, by his conservatism and continuous moist antiseptic dressing. The usual custom and fashion of the day would have sacrificed them in the effort to get a clean wound, healthy flaps, primary intention and secondary artificial limb.

---

### A WOMAN PROFESSOR OF ANATOMY.

Miss Florence R. Sabin has been appointed Associate Professor of Anatomy in the Johns Hopkins University. Her name is well known to anatomists by her work on the nervous system. Miss Sabin, it may be stated, is not the first woman who has been professor of anatomy. In the sixteenth century Alessandra Giovanni taught that subject at Bologna, and Anna Manzolini occupied a chair of anatomy in the same university in the middle of the eighteenth century. Maria Pettracina was professor of anatomy in the University of Ferrara toward the end of the same century. She was the author of several works on a subject which is exciting great interest at present, namely, the physical education of children.—*Medical News, September 9th*

